# Molecular modelling and docking analysis of a modelled *trmB* protein from *Pseudomonas aeruginosa* with selected chemical compounds

**DOI:** 10.6026/973206300210657

**Published:** 2025-04-30

**Authors:** Sakshi Bhati, Imteyaz Qamar, Nagendra Singh

**Affiliations:** 1 Department of Biotechnology, University School of Biotechnology, Gautam Buddha University, Greater Noida, Uttar Pradesh - 201312, India

**Keywords:** *trmB*, homology modeling, virtual screening, molecular dynamics, protein-ligand interaction

## Abstract

Antimicrobial resistance (AMR) threatens global health with rising antibiotic-resistant infections, requiring novel antimicrobial targets. Therefore, it is of interest to report the molecular modeling and docking analysis *trmB* from *Pseudomonas aeruginosa* with suitable compounds. We show two compounds (5136 and 9865603) from the chemical library have optimal binding with the modelled *trmB* from *Pseudomonas aeruginosa* for further consideration.

## Background:

TRNA modifications are crucial post-transcriptional processes that enhance the stability, structure and functionality of TRNAs [1]. These modifications regulate accurate decoding, prevent ribosomal stalling and contribute to the overall fidelity of protein synthesis [2]. Bacteria rely on a diverse set of tRNA modifications to adapt to environmental stresses, including nutrient deprivation and oxidative damage. Among these, methylation at specific nucleotides plays a pivotal role in maintaining the translational efficiency and structural integrity of tRNA molecules [3]. This highlights the functional necessity of tRNA-modifying enzymes in bacterial physiology. TRNA (guanine-N7)-methyltransferase B (*trmB*), a SAM-dependent methyltransferase, catalyzes the methylation of guanine at position 46 (m7G46) in tRNAs. This modification contributes to the stabilization of the tRNA's elbow structure, which is vital for ribosome interaction [4]. The enzyme's dependency on S-adenosylmethionine (SAM) as a methyl donor emphasizes its biochemical specificity and regulatory potential. Structurally, *trmB* exhibits a Rossmann-like fold that anchors SAM and facilitates methyl group transfer to the target nucleotide [5]. Recent studies indicate that *trmB* activity is not restricted to translation fidelity but extends to the regulation of oxidative stress response genes, such as katA and katB, underscoring its dual role in bacterial survival and adaptation [6]. *Pseudomonas aeruginosa* is a notorious opportunistic pathogen characterized by its intrinsic and acquired resistance to multiple antibiotics [7]. Resistance mechanisms include efflux pump overexpression, biofilm formation and enzymatic degradation of antibiotics [8]. Emerging evidence suggests a potential link between tRNA modifications mediated by *trmB* and antibiotic resistance in *P. aeruginosa*. Specifically, *trmB*-modified tRNAs influence protein synthesis under antibiotic-induced stress, possibly contributing to the pathogen's resilience [9]. Furthermore, the regulatory role of *trmB* in oxidative stress response highlights its broader involvement in the survival mechanisms of antibiotic-resistant bacteria [10]. Thus, *trmB* is a promising target for novel antimicrobial strategies.

*trmB*, a SAM-dependent methyltransferase, is central to tRNA modification and is implicated in maintaining translational fidelity under stress conditions in *Pseudomonas aeruginosa* [5]. mediated by *trmB*, such as m7G46, enhance tRNA stability, ensuring efficient protein synthesis even in challenging environments like antibiotic exposure [6]. Emerging studies suggest that targeting tRNA-modifying enzymes can impair bacterial fitness and reduce resistance by disrupting their adaptive mechanisms [3]. By inhibiting *trmB* activity, it may be possible to weaken *P. aeruginosa's* resilience to antibiotics, providing a novel pathway for addressing multidrug resistance. *In silico* studies have become indispensable in drug discovery due to their ability to streamline and accelerate the identification of potential therapeutic compounds. Computational techniques like molecular modeling, docking and molecular dynamics (MD) simulations allow for detailed insights into protein-ligand interactions, minimizing the need for initial costly and time-consuming experimental screenings [11]. Additionally, virtual screening of large chemical libraries, such as the PubChem database, enables the identification of promising analogs of natural substrates like SAM, which can be further analyzed for inhibitory potential [12]. Therefore, it is of interest to report the molecular modeling and docking analysis *trmB* from *Pseudomonas aeruginosa* with suitable compounds for further consideration.

## Methodology:

## Homology modelling:

The amino acid sequence of *trmB* was retrieved from the UniProt database. UniProt is a comprehensive resource for protein sequence and functional information, widely used for biological studies [13]. Homology modelling was performed to forecast the three-dimensional conformation of *trmB*, given the absence of an empirically resolved structure. The *trmB* sequence underwent a BLAST search against the Protein Data Bank (PDB) to discover appropriate templates exhibiting high sequence similarity and resolution. Templates exhibiting a minimum of 30% sequence identity and possessing high structural quality, as assessed by R-factors and resolution, were given precedence. Model development was conducted utilising swiss-model and modeller, two reputable platforms for comparative protein structure prediction. swiss-model offered an automated pipeline, but modeller facilitated manual refinement to enhance structural precision. The final model included significant functional residues, such as SAM-binding sites, derived from alignment with template structures. The template protein selected for modeling was Q02U31.1.A tRNA (guanine-N (7)-)-methyltransferase, which shared 50% sequence identity. The quality of the modeled *trmB* structure was validated using procheck v3.5 [14] to assess the Ramachandran plot, which examined backbone dihedral angles to confirm structural geometry. Additional virtual screening available pockets were done through Drug Rep server using default parameter to find binding pockets in protein. Additionally, the ConSurf server was employed to analyze the evolutionary conservation of the tRNA (methyltransferase B) (*trmB*) protein from *Pseudomonas aeruginosa*. Multiple sequence alignment (MSA) was constructed using the MAFFT algorithm integrated within the ConSurf pipeline. Conservation scores were mapped onto the *trmB* protein structure, modeled using homology modeling tools [15].

## Docking preparation of macromolecule (protein) and ligands:

## Preparation of macromolecule (protein):

The Protein PDB structure was opened and polar hydrogen atoms were added. Charges (Kollman and Gasteiger) were subsequently added and the charges were neutralized. The edit option was then used to select atoms and assign AD4 parameters. This file was saved as a Protein pdbqt file.

## Preparation of ligand:

PubChem database was queried to identify potential SAM analogs. Structural similarity between compounds was evaluated using the Tanimoto coefficient, which quantifies the degree of similarity between molecular fingerprints [16]. Only compounds with a Tanimoto score ≥ 0.85 to SAM were selected for further study, ensuring close structural resemblance and potential for effective competitive binding to *trmB* (Table 1). The ligand.pdb file was opened, torsions for the ligand were set and the file was saved as a ligand pdbqt file.

## Preparation of GPF (grid parameter file):

The protein pdbqt file and ligand file were opened. Grid parameters were configured to prepare the grid box such that it covered all the active sites previously identified. The grid dimensions were set to 60 x 60 x 60 in the x, y and z coordinates, respectively, with a spacing of 0.375 Å to ensure active site coverage. The file was saved as a grid.gpf file.

## Preparation of DPF (docking parameter file):

The protein pdbqt file and the ligand file were opened and all default parameters were accepted. The file was saved as a dock.dpf file using the Lamarckian genetic algorithm.

## Preparation of GLG and DLG files:

GLG and DLG files were generated using "Cygwin" with the following commands:

[1] For the GLG file: ./autogrid4.exe -p grid.gpf -l protein.glg

[2] For the DLG file: ./autodock4.exe -p dock.dpf -l protein.dlg

## Docking simulation:

Molecular docking was performed using software such as Auto Dock Tools, to simulate the interaction of each ligand with the protein. The docking was carried out using predefined parameters and a scoring function to evaluate the binding affinity of each ligand. The results included docking scores and the predicted binding poses of the ligands in the active site.

## Molecular dynamics simulation system settings:

To perform molecular dynamics (MD) simulations, the Schrödinger suite was utilized to evaluate the stability and dynamic behavior of the ligand-receptor complex identified through docking studies. The protein-ligand complex was first pre-processed using the Protein Preparation Wizard [17], which involved the addition of missing residues, assignment of protonation states and optimization of hydrogen bonding networks. The prepared system was then embedded into a TIP3P explicit water model in an orthorhombic box, ensuring a buffer distance of 10 Å from the protein surface [18]. Counter ions were added to neutralize the system and 0.15 M NaCl was introduced to mimic physiological conditions. The Desmond MD engine was employed to perform the simulations [19]. Energy minimization was carried out to remove steric clashes, followed by a thermal equilibration phase under an NPT ensemble at 300 K and 1 at m pressure for 1 ns. A production run of 100 ns was conducted, with a time step of 2 f s, recording the trajectory to analyze root-mean-square deviation (RMSD), root-mean-square fluctuation (RMSF) and hydrogen bonding patterns to evaluate the stability and conformational changes of the protein-ligand complex.

## Results:

## Homology modelling and structure validation of *trmB* protein:

The model generation was carried out using SWISS-MODEL and MODELLER, with SWISS-MODEL providing an automated pipeline and MODELLER allowing for manual refinements [20, 21]. Critical functional residues, including SAM-binding sites, were incorporated into the final structure based on sequence alignment with the selected templates (Q02U31.1.A tRNA (guanine-N (7)-)-methyltransferase). The modeling process successfully generated a *trmB* model based on identified templates with high sequence similarity (50%) and structural quality. The generated model underwent rigorous validation. The Ramachandran plot from PROCHECK confirmed that most residues were within favored regions, reflecting good stereochemical quality [14]. The quality of the homology model was assessed using Ramachandran plot statistics, as shown in the results. Out of a total of 244 residues, the majority fall within acceptable conformational regions, confirming the model's structural reliability. The structural stability and functional relevance of the *trmB* protein model were further validated based on the binding compatibility of SAM-binding sites. A total of five pockets were identified, ranked based on their volume, center coordinates and size. Based on comparative benchmarks (118 high-resolution structures with R-factors ≤20%), the model's 88.3% residues in most favored regions indicate good structural quality. Although slightly below the expected threshold of 90%, the low proportion of residues in disallowed regions (0.5%) confirms that the homology model is of high reliability and suitable for further studies such as molecular docking or dynamics simulations as summarized in the Table 2 & Figure 1 (see PDF). Additionally, the conservation scores obtained from the conserve server provide a relative measure of evolutionary conservation at each sequence site of the target chain. Residues are categorized by conservation scores ranging from 1 (variable) to 9 (highly conserved). Most of the residues in the sequence exhibit moderate to high conservation (scores of 4-9), indicating their potential functional or structural significance. Highly conserved regions (scores 8-9), marked in purple, are dispersed throughout the sequence, suggesting evolutionary constraints likely due to their roles in maintaining enzymatic activity or structural integrity. Conversely, less conserved residues (scores 1-3), marked in green, are scattered and may correspond to regions less critical for protein function or structural stability. These insights suggest that the conserved regions are likely involved in key catalytic or binding activities of the tRNA methyltransferase B, aligning with the sequence's functional importance (Figure 2 - see PDF).

## Virtual screening against *trmB* protein:

Virtual screening was performed to identify potential ligands that bind effectively to the active site of the *trmB* protein. Total 20 molecules were screened and to validate the screening and redocking protocol, we performed a control experiment using a known bound ligand, S-adenosylmethionine. The docking of the ligand was carried out and the best binding energy scores were recorded and analyzed (Table 1). Among the screened compounds, the one with the highest binding energy was compound 9865603, exhibiting a score of -7.59 kcal/mol demonstrated consistent binding affinity across its cluster. The second-best compound was molecule 5136; with a binding energy score of -7.38 kcal/mol. Molecule 9865603 appeared in 1 cluster with 1 pose, indicating that the docking result is highly consistent and stable. In contrast, molecule 5136 was represented in 2 poses within 1 cluster, suggesting some variability in the binding orientations. Molecule 5136 formed 5 hydrogen bonds with residues such as GLU 99, ASP 126, SER 27 and LEU 128. Hydrogen bonding improves specificity and stability of binding, residues like PHE 28 exhibit Pi interactions, critical for stabilizing aromatic ring systems in the ligand. Furthermore, ARG 31 and ASP 149 participate in ionic or polar interactions, strengthening ligand binding. Notable interacting residues include VAL 100, ILE 161 and ALA 127, which are involved in hydrophobic interactions with the ligand, enhancing its fit within the binding site (Figure 3 (see PDF), Upper panel). In case of molecule 9865603 which formed 4 hydrogen bonds such as ARG 31, GLU 99, ALA 127 and ASP149. Furthermore, ALA A: 127, ILE A: 75, VAL A: 100, HIS A: 101 and VAL A: 98 are involved in various van der Waals and other interactions improving ligand-protein hydrophobic contacts. ARG A: 31 participate in an attractive charge interaction, stabilizing the ligand via ionic forces. (Figure 3 (see PDF), down panel). These compounds were prioritized for further molecular dynamics (MD) simulations. Top 10 compounds with their binding scores were given in Table 3.

## MD Simulation of *trmB*:

Molecular dynamics (MD) simulations were conducted to evaluate the dynamic behavior, stability and interactions of the *trmB* protein in complex with compounds 5136 and 9865603 over a simulation time of 100 ns. Compound 5136: The protein-ligand complex showed consistent stability throughout the simulation, with an average Root Mean Square Deviation (RMSD) of 13.5 Å, indicating minimal structural fluctuations. The Root Mean Square Fluctuation (RMSF) analysis highlighted low residue-level flexibility, particularly around the active site, suggesting stable ligand binding. The Radius of Gyration (Rg) value remained stable at approximately 4.8 Å, confirming the compactness of the protein structure (Figure 4 - see PDF). Protein-ligand interactions can be explored through the 'Simulation Interactions Diagram' panel. The stacked bar charts are normalized over the course of the trajectory (Figure 5 - see PDF). Compound 9865603: This complex also demonstrated high stability, with an average RMSD of 13.5 Å. Although slightly higher than compound 5136, the deviations were well within an acceptable range. The RMSF analysis indicated moderate flexibility in some loop regions but stable interactions in the active site. The hydrogen bond analysis revealed 4 hydrogen bonds that persisted during the simulation. The Rg value remained stable around 4.75 Å, confirming the structural integrity of the protein-ligand complex (Figure 6 - see PDF). Protein-ligand interactions can be explored through the 'Simulation Interactions Diagram' panel. The stacked bar charts are normalized over the course of the trajectory (Figure 7 - see PDF). Overall, both compounds displayed favourable dynamic behavior and stable interactions within the *trmB* active site. However, compound 5136 exhibited slightly better stability and stronger binding, as evidenced by lower RMSD and a higher number of persistent hydrogen bonds. These findings suggest that both compounds are promising candidates for further optimization and experimental validation.

## Discussion:

The current study focused on the structural characterization of *trmB* protein using homology modeling, virtual screening and molecular dynamics (MD) simulations to identify potential ligands with strong binding affinity and stability. The results obtained provide critical insights into the structural features and functional dynamics of *trmB*, as well as the identification of promising compounds that could serve as potential inhibitors or modulators. The homology modeling of the *trmB* protein revealed a well-defined 3D structure, which enabled further structural and functional investigations. The top-ranked binding pockets, identified based on volume and spatial dimensions, indicated potential active sites for ligand interactions. Pocket 1, with the largest volume (3144 Å^3^) and dimensions (30, 21 and 25), was identified as the primary site of interest for ligand docking due to its size and accessibility. This observation aligns with previous studies on proteins of similar function, where the largest binding pocket often serves as the most favorable site for ligand binding [22]. The virtual screening process identified two lead compounds, 5136 and 9865603, with the best binding energies, indicating their high potential for interaction with the *trmB* active site. Compound 5136 exhibited a particularly strong affinity, consistent with findings in earlier studies where compounds with favorable binding energy typically form stable complexes with their target proteins [23]. Such virtual screening approaches have been successfully employed in prior studies to identify lead candidates against various bacterial proteins, demonstrating the efficacy of computational tools in early-stage drug discovery [21]. The molecular dynamics simulation results further validated the stability and dynamic behavior of the two protein-ligand complexes. Both compounds demonstrated low Root Mean Square Deviations (RMSD) and Root Mean Square Fluctuations (RMSF), confirming their stable binding within the active site. Compound 5136 displayed an average RMSD of 13.5 Å with consistent hydrogen bond interactions (5 bonds), indicating strong stabilization within the protein pocket. On the other hand, compound 9865603 exhibited slightly higher RMSD (13.5 Å) and moderate flexibility but maintained persistent hydrogen bond interactions (4 bonds). These results corroborate findings from previous MD simulation studies, which emphasize the importance of persistent hydrogen bonds and low RMSD values as indicators of protein-ligand complex stability [24, 25]. Additionally, the Radius of Gyration (RG) values for both complexes remained stable (4.75-4.8 Å), suggesting no significant structural disruption or unfolding during the simulation. The identification of stable protein-ligand interactions aligns with the results of studies conducted on other bacterial regulatory proteins, where ligands exhibiting strong hydrogen bond interactions and low flexibility were shown to inhibit protein function effectively [26]. The results also highlight the importance of molecular mechanics-based energy calculations, such as MM-PBSA and MM-GBSA, which have been successfully applied to predict ligand binding free energy in various protein systems [23].

## Conclusion:

Molecular modeling and docking analysis shows that two identified compounds (5136 and 9865603) from the chemical library have optimal binding with the modelled *trmB* from *Pseudomonas aeruginosa* for further consideration.

## Statements and declarations:

## Competing interests:

The authors declare no conflict of interest related to this study.

## Funding statement:

Study is funded by Indian Council of Medical Research, ICMR and India

## Figures and Tables

**Table 1:** Selected top compound list for the virtual simulations

**Sr. No.**	**Name of Compounds**	**Compound PubChem Id**
1	[Methyl-3H]-S-adenosyl-L-methionine	24762163
2	carboxy-S-adenosyl-L-methionine	90659115
3	(2R,3R,4R,5S)-2-(6-aminopurin-9-yl)-5-(methylsulfanylmethyl)oxolane-3,4-diol	453683
4	(2S)-2-amino-4-[[5-(6-aminopurin-9-yl)-3,4-dihydroxyoxolan-2-yl]methylsulfanyl]butanoic acid	59170628
5	2-amino-4-[[(2S,3S,4R,5R)-5-(6-aminopurin-9-yl)-3,4-dihydroxyoxolan-2-yl]methylsulfanyl]butanoic acid\	13792
6	2-amino-4-[[5-(6-aminopurin-9-yl)-3,4-dihydroxyoxolan-2-yl]methyl-(111C)methylsulfonio]butanoate	450572
7	[(3S)-3-amino-3-carboxypropyl]-[[5-(6-aminopurin-9-yl)-3,4-dihydroxyoxolan-2-yl]methyl]-methylsulfanium	71299730
8	2-amino-4-[[5-(6-aminopurin-9-yl)-3,4-dihydroxyoxolan-2-yl]methyl-methylsulfonio]butanoate	5136
9	S-adenosylmethionine	34756
10	[(3S)-3-amino-3-carboxypropyl]-[[(2S,3S,4R,5R)-5-(6-aminopurin-9-yl)-3,4-dihydroxyoxolan-2-yl]methyl]-(114C)methylsulfanium;chloride	11112916
11	[(3S)-3-amino-3-carboxypropyl]-[[(2S,3S,4R,5R)-5-(6-aminopurin-9-yl)-3,4-dihydroxyoxolan-2-yl]methyl]-methylsulfanium;sulfate	127256389
12	5'-Methylthioadenosine	439176
13	S-adenosyl-L-homocysteine	439155
14	Adenosine	60961
15	5'-S-[(3S)-3-azaniumyl-3-carboxylatopropyl]-5'-thioadenosine	25246222
16	S-Adenosylmethionine chloride	90473
17	S-adenosyl-L-methionine	24762165
18	5'-S-[(3S)-3-azaniumyl-3-carboxylatopropyl]-5'-thioadenosine	25246222
19	S-Adenosyl-DL-methionine	9865603
20	S-adenosylmethionine	34756

**Table 2:** Predicted binding pocket analysis during homology modelling

**Pocket**	**Volume**	**Center**	**Size**
1	3144	0.6, 8.7, 2.9	30, 21, 25
2	203	-2.4, 14.3, -4.9	13, 13, 13
3	104	-3.9, 8.0, -15.8	16, 12, 9
4	100	-9.0, -4.8, -8.8	12, 14, 11
5	63	9.2, 10.6, -16.1	12, 10, 10

**Table 3:** Below is a list of the top 10 compounds with their respective binding scores in kcal/mol during molecular docking

**S. No.**	**Compound PubChem Id**	**Mean Binding Energy**	**Numbers in clusters**	**h Bond**
1	9865603	-7.59	1	4
2	5136	-7.38	2	5
3	24762163	-7.46	1	5
4	127256389	-6.93	2	4
5	439155	-6.5	3	4
6	13792	-6.61	2	1
7	34756	-6.55	4	6
8	71299730	-6.38	3	3
9	25246222	-7.21	1	5
10	450572	-6.14	4	4
